# Sensor Location Problem Optimization for Traffic Network with Different Spatial Distributions of Traffic Information

**DOI:** 10.3390/s16111790

**Published:** 2016-10-27

**Authors:** Xu Bao, Haijian Li, Lingqiao Qin, Dongwei Xu, Bin Ran, Jian Rong

**Affiliations:** 1 Key Laboratory for Traffic and Transportation Security of Jiangsu Province, Huaiyin Institute of Technology, Huai’an 223003, China; baoxu@hyit.edu.cn; 2 Beijing Key Laboratory of Traffic Engineering, Beijing University of Technology, Beijing 100124, China; jrong@bjut.edu.cn; 3 Department of Civil and Environmental Engineering, University of Wisconsin-Madison, Madison, WI 53706, USA; lingqiao.qin@wisc.edu (L.Q.); bran@wisc.edu (B.R.); 4 College of Information Engineering, Zhejiang University of Technology, Hangzhou 310014, China; dongweixu@zjut.edu.cn

**Keywords:** traffic information engineering, traffic flow information, sensor location problem, optimization model, information spatially measure

## Abstract

To obtain adequate traffic information, the density of traffic sensors should be sufficiently high to cover the entire transportation network. However, deploying sensors densely over the entire network may not be realistic for practical applications due to the budgetary constraints of traffic management agencies. This paper describes several possible spatial distributions of traffic information credibility and proposes corresponding different sensor information credibility functions to describe these spatial distribution properties. A maximum benefit model and its simplified model are proposed to solve the traffic sensor location problem. The relationships between the benefit and the number of sensors are formulated with different sensor information credibility functions. Next, expanding models and algorithms in analytic results are performed. For each case, the maximum benefit, the optimal number and spacing of sensors are obtained and the analytic formulations of the optimal sensor locations are derived as well. Finally, a numerical example is proposed to verify the validity and availability of the proposed models for solving a network sensor location problem. The results show that the optimal number of sensors of segments with different model parameters in an entire freeway network can be calculated. Besides, it can also be concluded that the optimal sensor spacing is independent of end restrictions but dependent on the values of model parameters that represent the physical conditions of sensors and roads.

## 1. Introduction

An important mission of intelligent transportation systems is to build and extend traffic sensor networks to improve the transportation system’s observability, productivity, and efficiency [1]. Some studies have noted [2,3,4] that, with the advent of intelligent transportation systems, sensors, especially wireless sensors [5,6], are becoming increasingly critical elements of the modern cities and transportation systems. This growing need for real-time traffic information has resulted in an interesting class of problems collectively known as sensor location problems (SLP) [2]. According to [7], another important function of intelligent transportation systems is the collection of real-time traffic information, which is performed by traffic sensors, including vehicle counting sensors, traffic speed sensors, and other types of traffic sensors. In recent years, sensor technologies (e.g., loop detectors, surveillance cameras, travel time sensors, microwave sensors, and magnetic sensors) have been widely used on highway networks and urban arterial roads to estimate real-time traffic states and acquire real-time traffic information, which is valuable for both private agencies and public sectors. Real-time traffic information enables road users to choose the best routes and enables traffic managers to promptly respond to congestion patterns and efficiently select control strategies [8]. The effectiveness of intelligent transportation systems depends not only on the accuracy of the traffic information but also on the coverage over the road network [9]. To obtain adequate traffic information, the density of traffic sensors should be sufficiently high to adequately cover the entire transportation network. Properly locating traffic sensors is critical to the accurate estimation of real-time traffic status over transportation networks. If sensors are densely deployed over a transportation network and all of them collect real-time traffic data around their locations, the preferable estimation for the entire network can be obtained by each individual sensor using feasible algorithms. Moreover, abnormal traffic states (e.g., traffic accident detection and traffic congestion detection) can be detected [8].

The assumption of a network-wide traffic sensor system may not be realistic for practical applications due to the budgetary constraints of traffic management agencies [10]. Hu et al. [10] suggested that deploying a large number of sensors in an urban network of moderate size can involve substantial costs. Because the quantity and quality of the collected traffic flow information significantly affects the estimation accuracy and reliability of traffic flow on the entire network, there is a trade-off between the prediction accuracy of network traffic flow estimates and the cost associated with the extent to which a sensor system is deployed. Considering the rapid development of intelligent transportation systems in modern cities, it is common to see conflicts arising between the density requirement of fixed traffic sensors and the budget limit [7]. These conditions motivate the need to address the optimization problems involving locations and numbers of sensors given a limited budget.

Gentili and Mirchandani [11] addressed the models, challenges, and research opportunities of locating sensors on traffic networks and proposed that existing models differ according to different criteria: (1) sensor types to be located on the network; (2) availability of a priori information; and (3) flows of interest (e.g., O-D flows, route flows, and link flows). They reviewed the existing contributions and provided a unifying picture of these models by categorizing them into two main problems: the sensor location flow-observability problem and the sensor location flow-estimation problem. In past years, many researchers have addressed the SLP based on these two main problems, and sensors were optimally located for a central purpose of origin-destination (O-D) matrix estimation [1,2,8,9,10,12,13,14,15,16,17,18,19,20,21,22] or other flow issues [23,24,25,26,27,28,29,30]. A majority of researchers employ graph theory to solve the problem of O-D estimation. They transform this problem into a problem in graph theory and apply results from this field to obtain a solution [1,16,21,31,32]. The road network is modeled as a directed graph, with vertices representing intersections and directed edges between the vertices representing roads. The traffic flow over roads is described by a network flow function on the edges of the graph. Therefore, the goal of the problem is to find the smallest subset of edges such that knowledge of the flow along these edges uniquely determines the flow everywhere on the graph [16]. Yang et al. [33] proposed two critical problems: (1) how to select the optimal locations of a given number of counting stations to separate as many O-D pairs as possible; and (2) how to determine the minimum number of counting stations and their locations required for separating all O-D pairs. The problems of interest were formulated as integer linear-programming models, and a branch-and-bound technique was developed to find an optimal counting location solution. Ng [2] presented a reformulation of this link observability problem requiring only node enumeration and proved a conjecture from [10] by deriving an explicit relationship between the number of nodes and links in a transportation network and the minimum number of sensors to install to be able to infer all link flows. Some traffic speed-based methods were proposed as well to address the SLP, and they were mostly applied to estimate travel time [34,35,36,37]. Kima et al. [34] presented an approach that optimizes the location of sensors along a freeway to support more accurate estimations of travel times than those obtained from conventionally deployed fixed point sensors. In [37], a bilevel programming model was proposed for a network with travel time information. The lower-level problem was a probit-based traffic assignment model, whereas the upper-level problem involved determining the speed detector density that minimizes the measured travel time error variance and the social cost of the speed detectors.

In summary, those existing methods and models can solve the problems for some special applications (O-D estimate, travel time estimate), but they cannot address the SLP using all of the traffic information. As noted in [10], existing methods have been typically addressed as a sub-problem of broader problems related to O-D demand estimation, time-dependent link travel time estimation, or operational consistency-seeking procedures. Moreover, the following conditions should be considered: (1) Since there are many assumptions in graph theory, the results provide only the smallest subset of edges. Such methods typically assume that the availability of the turning proportions at a node or link use a proportion matrix that captures the proportion of O-D trips of a given path that traverse a specific link [10,21,22]. Moreover, the results will always involve identifying which edges should deploy sensors, but the detailed location of each sensor (the middle, the front end, or other parts of the edge) is not clear. (2) In the network, the weightiness at different locations might be different; hence, the weights of different locations should be considered in the SLP models. (3) As these sensor technologies continue to become more reliable and more cost effective, the demand for travel information is also growing. In some cases, more than one sensor (if there are several sensors in a section of an edge, they will be considered as one sensor here) will be located in a segment. (4) The information derived from fixed sensors in a traffic network will present different spatial distributions under different conditions (e.g., different types of road, different locations, and different types of sensors).

Without any mathematical model, Fujito et al. [38] conducted an empirical study to address the impact of sensor spacing along freeway corridors on the computation of a performance measure called the travel time index. Their results indicated that when sensors are deleted relative to the baseline sensor spacing condition, the congestion measure statistic will be overestimated under some spacing conditions and underestimated under others. Their empirical study illustrates the effect of sensor spacing on the calculation of corridor congestion measures, such as the travel time index. The paper also noted that if the objective is to estimate a metric that presents a comprehensive picture for the entire corridor, a denser sensor spacing is preferable. Selection of specific placement of the sensors is a key element in obtaining valid measures of corridor congestion. However, the optimal sensor spacing still had not been obtained in [38], and as they recommended that more work on the theoretical underpinnings of this study must be performed.

The aim of this paper is to address the sensor location problems with different spatial distributions of traffic information and to propose a theoretical method for optimizing the numbers and locations of traffic sensors for traffic networks. The remainder of the paper is organized as follows. Section 2 introduces the definition of traffic information credibility, presents three typical options of sensor information credibility functions (SICFs) and introduces the maximum benefit model and its simplified model. Section 3 focuses on the expanding models and algorithms based on maximum benefit model with different SICFs. Next, a numerical example is proposed to verify the solving methods for a network SLP in Section 4. Finally, Section 5 provides conclusions and application prospects.

## 2. Typical Sensor Information Credibility Functions and Optimization Models

### 2.1. Spatial Heterogeneity of Traffic Information Credibility

Existing methods of traffic information acquisition may depend on the distribution of sensor networks along roads. Those sensors, such as loop, magnetic, video, and microwave sensors, are always placed at fixed locations or in some micro-regions. The coverage area of such a single sensor is always a single- or multi-lane section.

For some traffic information acquisition systems, the unit of information acquisition area is almost a section of one-way road, and the sensors used in those systems can obtain information for all of the lanes. In this paper, the unit of information acquisition area is set as a section of one-way road, and the sensors in one section will be called sectional sensors (denoted simply as “sensor” in the following text). Given the topologies of road networks, the roads are regarded as directed line segments, and the intersections that connect those roads are regarded as vertices (network nodes).

As shown in [39], the essence of fixed-sensor-based information acquisition is to obtain the traffic information at some key points along the road, which are limited points for the entire road network. The traffic information for other places where there are no sensors cannot be acquired directly but can be estimated by the information from these limited points. For the phenomenon of fixed location or micro-region distributions of traffic information derived from fixed sensors, the traffic information credibility in the entire road network is heterogeneous. To describe the different spatial distributions of traffic information credibility, some SICFs have been proposed based on reasonable assumptions. (1) In a road with limited length, the traffic conditions at an upstream location and a downstream location have some causality. In other words, the traffic conditions at different locations are not independent; (2) In a road, the traffic condition of each location (a road section or a lane section) can be estimated by other locations with a certain degree of accuracy; (3) Closer locations will have higher degrees of credibility to the interest point.

Drezner and Wesolowsky [40] had shown that the probability that an event is detected by a detector is a decreasing function of the distance. In the paper, they assumed a general probability function of distance π(*d*), which is a monotonically decreasing function of the distance. They also studied two probability functions in particular, namely, a signal decay function and an exponential decay function. Based on those reasonable assumptions above, Li et al. [30,41] described the spatial distributions of traffic information credibility. Here, similar to π(*d*) in [40], the SICF is proposed to represent the spatial distribution of traffic information credibility from fixed sensors. By denoting origin o as the coordinate of a sensor, the SICF can be formulated as:

(1) SICF=f(x) x∈(−∞,+∞),

where *x* is the distance away from a sensor by directivity. Because of the directivity of distance measure along a road, the value of *x* is positive if the direction away from the sensor is the right side of the one-way road; otherwise, the *x* is negative. ∀*x*, 0 ≤ *f*(*x*) ≤ 1 and *f*(0) = 1, *f*(∞) = 0. In practice, the definitional domain of *f*(*x*) will be the length of a study one-way road or corridor.

### 2.2. Typical Options of SICF

**Figure 1:**
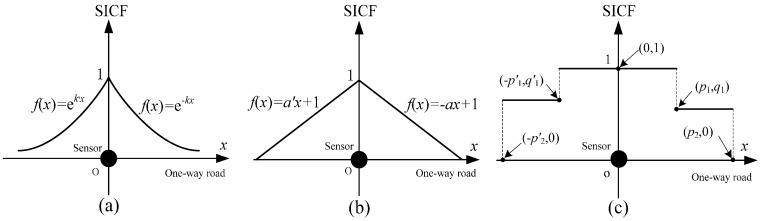
Three typical SICFs: (**a**) the EAF; (**b**) the LAF; and (**c**) the two-SAF.

SICF can represent different spatial distributions of traffic information credibility. Different micro-regions, such as ramp entrances, ramp exits, merging areas, or diversion areas, will have different spatial characteristics of traffic flow. One can conclude that the spatial distributions of traffic information credibility will be multiform. In fact, for different conditions of roads and sensors, there are various *f*(*x*) that can calibrate the SICF. As discussed in [41], three typical SICFs (exponential attenuation SICF (EAF), linear attenuation SICF (LAF), and step attenuation SICF (SAF)) are shown in Figure 1. Based on field data, Li et al. [30,41] have determined that the SICF can be calibrated using field data. Studying these three typical SICFs will be helpful to reveal more complex spatial distributions of traffic information credibility for actual road networks.

As shown in Figure 1a, the EAF represents a nonuniform gradient of traffic information credibility that can be formulated by an exponential function, which stands for an exponentially decreasing probability of detection (as described in [40]). As discussed in [30,41], *k*, *k*′, *a*, *a*′ and *p*_1_, *p*_2_, *p*′_1_, *p*′_2_, *q*_1_, *q*′_1_ are the coefficients of these three typical SICFs and the values of all these coefficients are positive. For more common situations, it should be noted that the left and right of vertical axis of a SICF may not be the same attenuation pattern and they may be a combination of two different attenuation functions.

### 2.3. Optimization Models of One-Way Road

For different detection regions (a single lane, multiple lanes, a road section, or multiple road sections) of the sensors used to acquire traffic information, in this paper, the base unit of the detection region is set as a one-way road section, i.e., a sensor at location *i* can detect the traffic information of all lanes in the section including location *i*. Unless otherwise noted, “sensor” refers to a sectional sensor of a one-way road. Here, the maximum benefit model [30,42] is introduced to optimize the SLP of one-way road. First, the model determines the locations of the first and last sensors in the one-way road (usually refers to the ends of the one-way road or the nodes in a traffic network). If the first and last locations cannot be determined, the starting and ending points of the one-way road will be selected as the preselected locations of the first and last sensors. Whether the two points are located by sensors depends on the engineering requirement. Denote *L* as the length of the one-way road; *n* locations (including the locations of the first and last sensors) are selected as the preselected locations that would be deployed by sensors. Those sensor locations are numbered as *i* (*I* = 1, 2, …, *n*). Denote the origin *o* as the location of the first sensor; then, the maximum benefit model can be formulated as:

(2) $$\max\;z=\sum_{i=1}^{n}B_{i}\times Q(i)\times V(i)\times \frac{\int_{a_{i-}}^{a_{i+}}f_{i}(x-x_{i})dx}{\int_{-\infty }^{+\infty }f_{i}(x-x_{i})dx}-\sum_{i=1}^{n}B_{i}C(i),$$

(3) $$s.t.\;\{\begin{array}{l} B_{i}=0\begin{array}{c} or \end{array}1\\ x_{i}=(i-1)d\\ d=L/(n-1) \end{array},$$

where *z* is the maximum benefit of all sensors located on the one-way road; *B_i_* is equal to 1 if a sensor is located at location *i* and 0 otherwise; *Q*(*i*) is the sensor accuracy of location *i*; *V*(*i*) is the benefit of traffic information of location *i*; *C*(*i*) is the sensor integration cost of location *i*; *x_i_* is the coordinate of location *i*; *d* is the distance of two adjacent candidate locations and *d* = *x_i_*_+1_ − *x_i_*; *f_i_*(*x*) is the SICF of sensor *i*; *a_i−_* and *a_i+_* are the superposition point coordinates of *f_i_*(*x*) and *f_i+_*(*x*), *f_i−_*(*x*), respectively; and *a*_1−_ = 0, *a_n_*_+_ = *L* (*f_i+_*(*x*) and *f_i−_*(*x*) are the SICFs of the left adjacent location *i−* with *B_i−_* = 1 and the right adjacent location *i*_+_ with *B_i_*_+_ = 1, respectively).

To simplify the maximum benefit model and make it easy to reveal the relationship between the optimal number of sensors and model parameters in theory, it is assumed that each model parameter has the same value at different locations. *f*(*x*), *V*, *C*, and *Q* is used to denote *f_i_*(*x*), information value *V*(*i*), integration cost *C*(*i*), and accuracy *Q*(*i*) at location *i*, respectively. Moreover, using the calibration results of SICF in [30,41], *f*(*x*) is a symmetrical function on the vertical axis, namely, *f*(*x*) = *f*(−*x*). In the superposition area of two SICFs, the larger SICF value at the superposition point is selected as the final SICF value (different spatial superposition patterns are referred in [41]. Then, from Equations (2) and (3), the simplified maximum benefit model can be expressed as:

(4) $$z(n)=\{\begin{array}{ll} \frac{\frac{QV}{2}\int_{0}^{L}f(x)dx}{\int_{0}^{+\infty }f(x)dx}-C & \mathrm{when}\;n=1\\ (n-1)\frac{QV\int_{0}^{d/2}f(x)dx}{\int_{0}^{+\infty }f(x)dx}-nC & \mathrm{when}\;n>1 \end{array},$$

Equation (4) indicates the relationship between the number of sensors (*n*) and the maximum benefit (*z*(*n*)). Additionally, the equation illustrates the changing trend of maximum benefit *z*(*n*) when *n* changes. According to the changing trend of *z*(*n*), the optimal number and spacing of sensors can be determined. When *n* = 1, the starting or ending point of the one-way road will be deployed by a sensor; when *n* > 1, both the starting and ending points will be deployed by sensors, which is required by the simplified maximum benefit model of one-way road.

## 3. Expanding Models and Algorithms with Different SICFs

The end restriction refers to whether the sensor is located at the starting or ending point of a one-way road. Two kinds of end restriction, which are fixation at both ends and freeness at both ends, will be discussed with different SICFs.

### 3.1. Exponential Attenuation SICF (EAF)

An EAF in the entire region will be:

(5) $$f(x)=\{\begin{array}{ll} e^{-kx} & x\in [0,+\infty )\\ e^{k^{\prime }x} & x\in (-\infty ,0) \end{array}.$$

If the EAF is a longitudinally symmetrical function, i.e., *k* = *k*′, then Equation (5) simplifies to: *f*(*x*) = *e*^−*k*|*x*|^.

#### 3.1.1. Case A: Fixation at Both Ends with EAF

**Figure 2:**
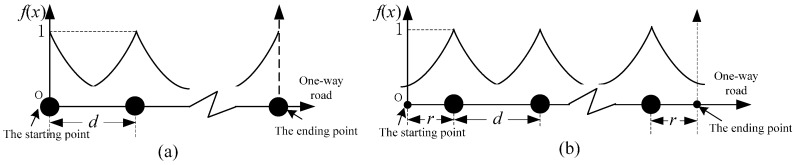
Sensor locations of the exponential attenuation when *n* > 1: (**a**) Case A; and (**b**) Case B.

The main features of Case A are that the starting and ending points are both fixed by a sensor and other sensors are located equidistantly between the starting and ending points. The sensor locations of Case A when *n* > 1 are shown in Figure 2a.

Based on Equation (4), the formula of the maximum benefit model of Case A is:

(6) $$z_{A}(n)=(n-1)QV(1-e^{-\frac{kL}{2(n-1)}})-nC.$$

where *z*_A_(*n*) is the benefit with *n* sensors evenly located for Case A.

*z*_A_(*n*) is a convex function and has a unique solution. It is difficult to obtain the analytic solution of Equation (6). Algorithm 1 can be adopted to find the solution (denote *z*_A*m*_ as the maximum benefit of the road for Case A). Given the optimal number of sensors *N*_A_, the optimal sensor spacing for a one-way road will be *d*_Aopt_ = *L*/(*N*_A_ − 1).

**Algorithm 1. Solving Equation (6)****Input:**Model parameters
*Q*, *V*, *C*, *L*, and *k***Output:***N*_A_, *z*_Am_1:**Initialization:**
*Q*, *V*, *C*, *L*, *k*; z_temp = 0; *z*_A*m*_ = 0; *n* = 2;2:Done = 1;3:**while** done4:  *z* = (*n* − 1) × *Q* × *V ×* (1 − exp(−0.5 × *k* × *L*/(*n* − 1))) 5:  – *n* × *C*;6:  **if**
*z* > z_temp7:    *n*++;8:    z_temp = *z*;9:  **else**10:    done = 0;11:  **end if**12:  **end while**13:*N*_A_ = *n*;
*z*_A*m*_ = z_temp.

#### 3.1.2. Case B: Freeness at Both Ends with EAF

Each model parameter is assumed to be the same at different locations, and due to the symmetry principle, the optimal sensor locations must be centrosymmetric and equidistant, as shown in Figure 2b. Denote *r* as the distance between the first (last) sensor and the start (end) point of a one-way road. When *n* sensors are located in a one-way road, *z*_B_(*n*) is formulated as:

(7) $$z_{B}(n)=(n-1)QV(1-e^{-\frac{k(L-2r)}{2(n-1)}})+QV(1-e^{-kr})-nC=QV((n-1)(1-e^{-\frac{k(L-2r)}{2(n-1)}})+(1-e^{-kr}))-nC,$$

where *z*_B_(*n*) is the benefit with *n* sensors evenly located for Case B.

Construct an assistant function *g*(*r*):

(8) $$g(r)=(n-1)(1-e^{-\frac{k(L-2r)}{2(n-1)}})+(1-e^{-kr}).$$

Select *r* as the independent variable in Equation (8). When *g*(*r*) has a maximum value, *z*_B_(*n*) can obtain the maximum value. Thus, letting *g*′(*r*) = 0, it can be obtained that $e^{-kr}-e^{-\frac{k(L-2r)}{2(n-1)}}=0$, i.e.,:

(9) $$-kr=-\frac{k(L-2r)}{2(n-1)},\;r=\frac{L}{2n}.$$

Substituting Equation (9) into Equation (8) yields:

(10) $$z_{B}(n)=nqV(1-e^{-\frac{kL}{2n}})-nC.$$

Similarly, the optimal number of sensors *N*_B_ and the maximum benefit of Case B *z*_B*m*_ can be obtained based on Equation (10) and Algorithm 1, and then the optimal sensor spacing will be:

*d*_Bopt_ = (*L−* 2*r*)/(*N*_B_ − 1) = *L*/*N*_B_. (11)

Specifically, when *n* = 1, it can be got *r* = *L*/2, then:

(12) $$z_{B}(1)=QV(1-e^{-\frac{kL}{2}})-C.$$

### 3.2. Linear Attenuation SICF (LAF)

LAF is a representation of the uniform gradient for traffic information credibility, which can be formulated by a linear function (Figure 1b). A LAF is given as:

(13) $$f(x)=\{\begin{array}{ll} -ax+1 & x\in [0,\frac{1}{a}]\\ a^{\prime }x+1 & x\in [-\frac{1}{a},0)\\ 0 & \mathrm{otherwise} \end{array},$$

If *a* = *a*′, Equation (13) simplifies to: *f*(*x*) = −*a*|*x*| + 1.

#### 3.2.1. Case C: Fixation at Both Ends with LAF

Because of the nonconstant parameter *a*, the SICF with a linear attenuation will be more complex than that with an exponential attenuation. Given the optimal number of sensors *N*_C_, the optimal sensor spacing will be *d*_Copt_ = *L*/(*N*_C_ − 1), and $\frac{2}{a}>d_{\mathrm{Copt}}$.

**Figure 3:**
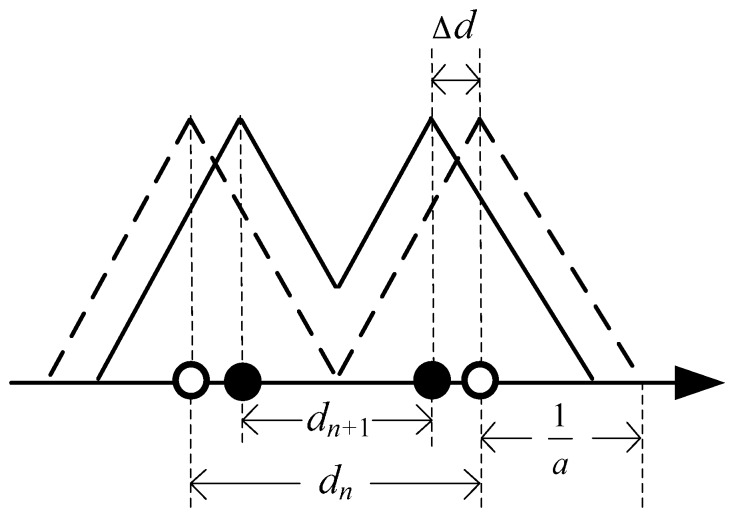
Sketch map of *d_n_*, *d_n_*_+1_, and 1/*a*.

**Proof.**  When *n* sensors are located in the road, then *d_n_* = L/(*n* − 1) and z(n)=(n−1)QV−nC. It is assumed that $\frac{2}{a}=d_{n}$. When another sensor is added to the road (Figure 3), *z*_C_(*n* + 1) is formulated as:

(14) $$z_{C}(n+1)=nQV(1-{\left(1-(a(\frac{1}{a}-\Delta d))\right)}^{2})-(n+1)C=nQV(1-{\left(a\Delta d\right)}^{2})-(n+1)C.$$

From those equations, $\Delta d=\frac{d_{n}-d_{n+1}}{2}$, $d_{n}=\frac{L}{n-1}$, $d_{n+1}=\frac{L}{n}$ and $a\Delta d=\frac{1}{n}$, then:

(15) $$z_{C}(n+1)-z_{C}(n)=(1-\frac{1}{n})QV-C.$$

From the field data, QV>>2C. For n≥2, the inequality $z_{C}(n+1)-z_{C}(n)>>0$ can be obtained from Equation (15). Therefore, it is better to locate *n* + 1 sensors than *n* sensors in the road. Now, it is demonstrated that $d_{n+1}<d_{n}=\frac{2}{a}$, i.e.,:

(16) $$d_{\mathrm{opt}}\leq d_{n+1}<d_{n}=\frac{2}{a}.$$

□

**Figure 4:**
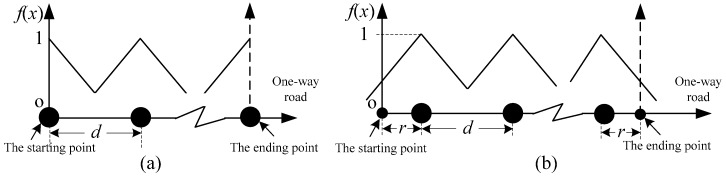
Sensor locations with a linear attenuation when *n* > 1: (**a**) Case C; and (**b**) Case D.

For $\frac{2}{a}>d_{\mathrm{opt}}$, the optimal sensor location strategy of Case C can be shown in Figure 4a, and *z*_C_(*n*) is formulated as:

(17) $$z_{C}(n)=(n-1)QV(1-{\left(1-\frac{ad}{2}\right)}^{2})-nC=aLQV-(\frac{a^{2}L^{2}QV}{4(n-1)}+nC),$$

where *z*_C_(*n*) is the benefit with *n* sensors evenly located for Case C.

Letting ${z^{\prime }}_{C}(n)=0$, *z*_C_ has the maximum value when $n=1+\frac{aL}{2}\sqrt{\frac{QV}{C}}$. Denote <*n*> as the ceiling or floor operation of *n* that makes *z*(<*n*>) be a maximum one, then $N_{C}=<1+\frac{aL}{2}\sqrt{\frac{QV}{C}}>$, and substituting this result into Equation (17) yields:

(18) $$z_{Cm}\approx aLQV-aL\sqrt{QVC}-C,$$

where *z*_C*m*_ is the maximum benefit of the road for Case C. 

#### 3.2.2. Case D: Freeness at Both Ends with LAF

Refer to the method of Case B, it is obtained that *d* = (*L* − 2*r*)/(*n* − 1). The sensor locations of Case D are shown in Figure 4b. Similarly, the formula of *z*_D_(*n*) can be expressed as:

(19) $$z_{D}(n)=(n-1)QV(1-{\left(1-\frac{ad}{2}\right)}^{2})+QV(1-{\left(1-ar\right)}^{2})-nC,$$

where *z*_D_(*n*) is the benefit with *n* sensors evenly located for Case D.

Simplifying Equation (19) yields:

(20) $$z_{D}(n)=aLQV-\frac{a^{2}L^{2}QV}{4(n-1)}-nC+\frac{a^{2}LQV}{n-1}r-\frac{na^{2}QV}{n-1}r^{2}.$$

Construct an assistant function *g*(*r*) and let

(21) $$g(r)=\frac{a^{2}LQV}{n-1}r-\frac{na^{2}QV}{n-1}r^{2}.$$

When *g*(*r*) has a maximum value, Equation (20) can be maximized. Set *g*′(*r*) = 0; then, $\frac{a^{2}LQV}{n-1}-\frac{2na^{2}QV}{n-1}r=0$, i.e., $r=\frac{L}{2n}$. Substituting this value into Equation (20) yields:

(22) $$z_{D}(n)=aLQV-(\frac{a^{2}L^{2}QV}{4n}+nC).$$

Letting ${z^{\prime }}_{D}(n)=0$, *z*_D_ achieves its maximum value when $n=\frac{aL}{2}\sqrt{\frac{QV}{C}}$.

Substituting $n=\frac{aL}{2}\sqrt{\frac{QV}{C}}$ into Equation (22) yields:

(23) $$z_{Dm}\approx aLQV-aL\sqrt{QVC},$$

where *z*_D*m*_ is the maximum benefit of the road for Case D.

Rounding *n* to obtain the optimal number of sensors of Case D, and $N_{D}=<\frac{aL}{2}\sqrt{\frac{QV}{C}}>$.

**Figure 5:**
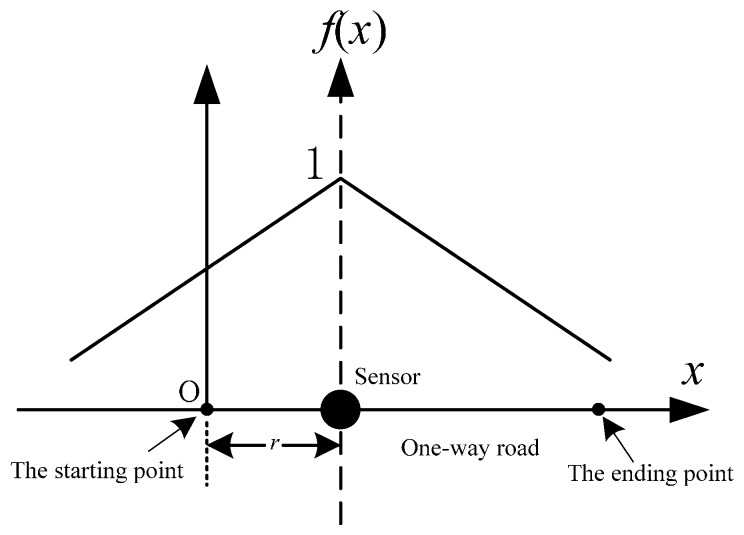
Sensor locations of Case D with a linear attenuation when *n* = 1.

Specifically, when *n* = 1, the sensor location can be shown in Figure 5.

If $\frac{2}{a}\geq L$, according to Equation (22), then:

(24) $$z_{D}(1)=aL(1-\frac{aL}{4})QV-C.$$

If $\frac{2}{a}<L$, for $x\in \left[\frac{1}{a},L-\frac{1}{a}\right]$, the maximum benefit can always be obtained, which is:

(25) $$z_{D}(1)=QV-C.$$

### 3.3. Two-Step Attenuation SICF (Two-SAF)

A two-SAF is shown in Figure 1c. The typical characteristic of a two-SAF is that the traffic information credibility will be stable in the area near the sensor but will suddenly decrease to another value and remain stable for a range of distances in a farther away area. The formula of a two-SAF is:

(26) $$f(x)=\{\begin{array}{ll} 1 & x\in [-{p^{\prime }}_{1},p_{1}]\\ q_{1} & x\in (p_{1},p_{2}]\\ {q^{\prime }}_{1} & x\in [-{p^{\prime }}_{2},-{p^{\prime }}_{1})\\ 0 & \mathrm{otherwise} \end{array}.$$

If the two-SAF is longitudinally symmetrical, namely, *p*_1_ = *p*′_1_, *p*_2_ = *p*′_2_, and *q*_1_ = *q*′_1_, then:

(27) $$f(x)=\{\begin{array}{ll} 1 & x\in [-p_{1},p_{1}]\\ q_{1} & x\in [-p_{2},-p_{1})\cup (p_{1},p_{2}]\\ 0 & \mathrm{otherwise} \end{array}.$$

#### 3.3.1. Case E: Fixation at Both Ends with SAF

According to the typical feature of the two-SAF and the restriction of *QV* >> *C*, the optimal sensor spacing *d*_E__opt_ meets the restriction of *p*_1_ ≤ *d*_E__opt_/2 ≤ *p*_2_. Therefore, if there are *n* sensors located in the road when *d* = *d*_E__opt_, then:

(28) $$1+\frac{L}{2p_{2}}\leq n\leq 1+\frac{L}{2p_{1}}.$$

**Figure 6:**
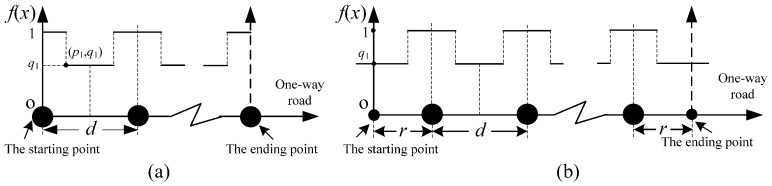
Sensor locations of the step attenuation when *n* > 1: (**a**) Case E; and (**b**) Case F.

Figure 6a presents the sensor locations of Case E when *n* > 1, and *z*_E_(*n*) will be formulated as:

(29) $$z_{E}(n)=(n-1)\frac{p_{1}+(\frac{d}{2}-p_{1})q_{1}}{p_{1}+(p_{2}-p_{1})q_{1}}QV-nC,$$

where *z*_E_(*n*) is the benefit with *n* sensors evenly located for Case E.

Substituting *d* = *L*/(*n* − 1) into Equation (29) yields:

(30) $$z_{E}(n)=\frac{\frac{q_{1}}{2}L-p_{1}+p_{1}q_{1}}{p_{1}+(p_{2}-p_{1})q_{1}}QV+\left(\frac{QV}{1+\frac{p_{2}q_{1}}{p_{1}-p_{1}q_{1}}}-C\right)n.$$

If $\varphi =\frac{qV}{1+\frac{p_{2}q_{1}}{p_{1}-p_{1}q_{1}}}-C\leq 0$, based on Equation (28), when $n=1+\frac{L}{2p_{2}}$, the maximum *z*_E_ will be obtained, and $N_{E}=<1+\frac{L}{2p_{2}}>$. Substituting this value into Equation (30) yields:

(31) $$z_{Em}\approx \frac{L}{2p_{2}}QV-(1+\frac{L}{2p_{2}})C,$$

where *z*_E*m*_ is the maximum benefit of the road for Case E.

If φ>0, based on Equation (28), when $n=1+\frac{L}{2p_{1}}$, *z*_E_ achieves its maximum value, and $N_{E}=<1+\frac{L}{2p_{1}}>$. Substituting this value into Equation (30) yields:

(32) $$z_{Em}\approx \frac{L}{2(p_{1}+(p_{2}-p_{1})q_{1})}QV-(1+\frac{L}{2p_{1}})C.$$

#### 3.3.2. Case F: Freeness at Both Ends with SAF

Similarly, with Case E, the optimal sensor spacing *d*_F__opt_ and *r* of Case F meets the conditions:

*p*_1_ ≤ *d*_F__opt_/2 ≤ *p*_2_. (33)

*p*_1_ ≤ *r* ≤ *p*_2_. (34)

Assume that the number of sensors located in a one-way road is close to the optimal number; then, the following relationship is obtained:

*d*_F__opt_ ≈ *d* = (*L* − 2*r*)/(*n* − 1). (35)

Compared with Equations (33) and (34), the restriction on *n* is:

(36) $$1+\frac{L-2r}{2p_{2}}\leq n\leq 1+\frac{L-2r}{2p_{1}}.$$

Then, *z*_F_(*n*) is formulated as:

(37) $$z_{F}(n)=(n-1)\frac{p_{1}+(\frac{d}{2}-p_{1})q_{1}}{p_{1}+(p_{2}-p_{1})q_{1}}QV+\frac{p_{1}+(x-p_{1})q_{1}}{p_{1}+(p_{2}-p_{1})q_{1}}QV-nC,$$

where *z*_F_(*n*) is the benefit with *n* sensors evenly located for Case F.

Substituting Equation (35) into Equation (37) yields:

(38) $$z_{F}(n)=\frac{q_{1}L}{p_{1}+(p_{2}-p_{1})q_{1}}QV+\left(\frac{QV}{1+\frac{p_{2}q_{1}}{p_{1}-p_{1}q_{1}}}-C\right)n.$$

From Equation (38), for *p*_1_ ≤ *r* ≤ *p*_2_, the value of *z*_F_(*n*) is not influenced by *r*.

If φ≤0, when $n=1+\frac{L-2r}{2p_{2}}$ (∀*r* for *p*_1_ ≤ *r* ≤ *p*_2_), the maximum *z_F_* is obtained. Let *r* = *p*_2_ (without loss of generality) as a special case; $n=\frac{L}{2p_{2}}$ when *d*_Fopt_ = 2*p*_2_ = 2*r*. The optimal number of sensors is $N_{F}=<1+\frac{L}{2p_{2}}>$, and the maximum benefit of Case F is

(39) $$z_{Fm}\approx \frac{L}{2p_{2}}QV+\frac{q_{1}L}{2(p_{1}-(p_{2}-p_{1})q_{1})}QV-\frac{L}{2p_{2}}C,$$

where *z*_F*m*_ is the maximum benefit of the road for Case F.

If φ≤0, when $n=1+\frac{L-2r}{2p_{1}}$ (∀*r* for *p*_1_ ≤ *r* ≤ *p*_2_), the maximum *z*_F_ is obtained. Let *r* = *p*_1_ (without loss of generality) as a special case; $n=\frac{L}{2p_{1}}$ when *d*_Fopt_ = 2*p*_1_ = 2*r*. The optimal number of sensors is $N_{F}=<\frac{L}{2p_{1}}>$, and the maximum benefit of Case F is

(40) $$z_{Fm}\approx \frac{(1+q_{1})L}{2(p_{1}+(p_{2}-p_{1})q_{1})}QV-(1+\frac{L}{2p_{1}})C.$$

**Figure 7:**
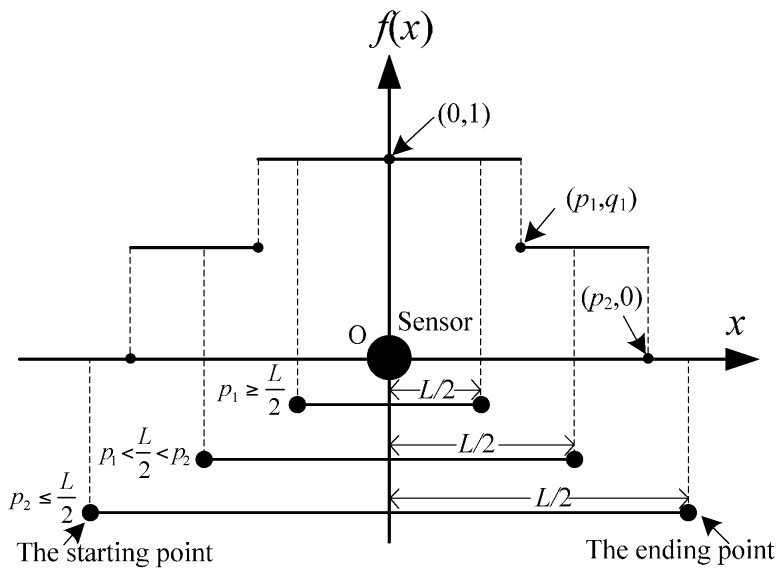
Sensor locations of a two-step attenuation when *n* = 1.

For φ≤0 or φ>0, when *z*_F_ is the maximum value *z*_F*m*_, *r* is a nonconstant value whose region is [*p*_1_, *p*_2_]. However, for the two special conditions, when *d* = 2*r*, *z*_F_ will always achieve its maximum value. Specifically, when *n* = 1, the maximum benefit will be obtained when the sensor is located in the middle of the one-way road (Figure 7).

According to the relationships between *L*/2 and *p*_1_ and *p*_2_, the maximum benefit *z*_F_(1) can be formulated as:

(41) $$z_{F}(1)=\{\begin{array}{ll} \frac{L}{2(p_{1}+(p_{2}-p_{1})q_{1})}QV-C & p_{1}\geq \frac{L}{2}\\ \frac{p_{1}+(\frac{L}{2}-p_{1})q_{1}}{p_{1}+(p_{2}-p_{1})q_{1}}QV-C & p_{1}<\frac{L}{2}<p_{2}\\ QV-C & p_{2}\leq \frac{L}{2} \end{array}.$$

### 3.4. Discussion

This work has mainly studied the situation involving a simplified scenario comparing with field traffic road networks using the simplified MBM. The values of each parameter of the simplified MBM at different locations are the same in this scenario. However, these models still yield some formulas and results that are beneficial for optimizing the SLP in practice. These models and results will also be helpful when studying more complex scenarios for the SLP in the future.

(1) Model parameters

These models have four basic parameters: the sensor accuracy *Q*, the sensor information benefit *V*, the sensor integration cost *C*, and the road length *L*. All of these parameters can be calibrated using the field data associated with the road and sensors selected for the SLP. Some calibration methods for those parameters have been studied in [30,41,42,43], and the values of these parameters are determined by field data in Beijing. In addition, some additional parameters should be calibrated for different SICFs, such as *k*, *a*, *p*_1_, *p*_2_, and *q*_1_. The parameters could been obtained through various fitting methods using field data [30,41]. Given these parameters, the maximum benefit, the optimal number (or spacing) of sensors, and the optimal sensor locations can be calculated by the corresponding models and formulas presented in Section 4.

(2) Optimal sensor locations

Given the optimal numbers of sensors for different Cases, Table 1 presents the formulas of optimal sensor locations for different SICFs (Denote *d_opt_* as the optimal distance between two adjacent sensors in the one-way road, *X_i_* as the optimal coordinate of the sensor *i* in the road). The formulas of optimal sensor locations are the same for same end restriction case, which indicates that the formula formats of the optimal sensor locations do not depend on the spatial distributions of traffic information credibility and only relate to the end restriction cases. Therefore, when the number of sensors (optimal number or planned number) is determined in engineering applications without knowing the spatial distributions, it is still possible to place the sensors at their optimal locations using these formulas. Table 1 also indicates that when *n* = 1, placing the sensor in the middle of the one-way road will be the optimal mode that can acquire the maximum benefit. When *n* = 1, the maximum benefit of different end restriction cases with the same SCIF are different. From Equations (6) and (10), the maximum benefit of Case B is always greater than that of Case A, i.e., $z_{Bm}(1)=QV(1-e^{-\frac{kL}{2}})-C$ is greater than $z_{Am}(1)=\frac{QV}{2}(1-e^{-kL})-C$. Equations (17) and (24) demonstrate that $z_{D}(1)>z_{C}(2)$ (when *C* > 0). Although the integration cost would be reduced to zero, which is $z_{D}(1)=z_{C}(2)$. Therefore, when there is only one sensor to be located on a one-way road, placing it in the middle will be the best choice. When *n* > 1, deploying sensors equidistantly will be an optimal mode if the values of each parameter at different locations are the same or close to a given value. Moreover, it can be determined that $z_{B}(N)>z_{A}(N)$, $z_{D}(N)>z_{C}(N)$, or $z_{F}(N)>z_{E}(N)$ from Equations (6), (10), (17), (22), (30) and (38). If the number of sensors is fixed, Case B, D or F will be the first layout mode. Similarly, from the LAF and SAF, it is concluded that $N_{C}+1\approx N_{D}$, $N_{E}+1\approx N_{F}$ and $z_{Dm}>z_{Cm}$, $z_{Fm}>z_{Em}$. It can be drawn that Case B, D or F require less number of sensors and acquire higher benefit. Furthermore, for different end restrictions, it can be determined that $d_{\mathrm{Aopt}}=d_{\mathrm{Bopt}}$, $d_{\mathrm{Copt}}=d_{\mathrm{Dopt}}$, $d_{\mathrm{Eopt}}=d_{\mathrm{Fopt}}$. Namely, the optimal sensor spacing is independent of end restrictions and is only dependent on the values of the model parameters, which represent the physical conditions of the sensors and roads.

(3) Maximum benefit and optimal number of sensors

The formulas of the maximum benefit and corresponding optimal numbers of sensors with different SICFs have been obtained. Except for EAF, the analytic formulas of the optimal number of sensors *N* and the maximum benefit *z_m_* can also be got, which can be calculated directly using these model parameters. For EAF, the analytic formulas of the benefit *z* and number of sensors *n* are obtained. The maximum surplus benefit *z_m_* and optimal number of sensors *N* can be easily determined based on Algorithm 1 for Case A and B.

## 4. Numerical Example

**Figure 8:**
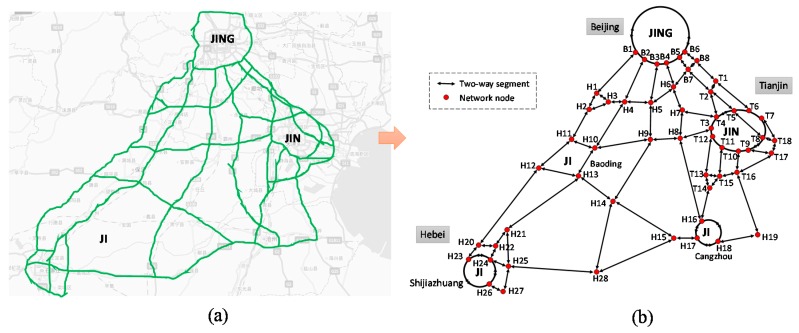
Freeway network of this numerical example: (**a**) the field freeway network; and (**b**) the topology of the freeway network.

A main part of freeway network in Beijing-Tianjin-Hebei region (Jing-Jin-Ji) is used to verify the validity of the models and algorithms proposed in this paper. As a national development strategy, the Chinese government has approved a plan to promote the transportation integration in the large Jing-Jin-Ji region. Then the demands of traffic monitoring and detecting in this region will be satisfied. The freeway network of this numerical example is shown in Figure 8a, which shows the main freeway corridors in the Jing-Jin-Ji region (B4 ↔ H6, B7 ↔ B8 and B7 ↔ H6 are planning segments). Based on Figure 8a, the corresponding network topology is shown in Figure 8b which contains 54 network nodes and 89 network links (two-way segments). For numerical computation, the basic information (network link index, name, length, etc.) and model parameters are shown in Table 2. The parameters of SICF with EAF, LAF and SAF are set as follows: *k* = 0.15, *a* = 0.10, *q*_1_ = 0.6, *p*_1_ = 0.4 and *p*_2_ = 1.2. In addition, the sensor accuracy is set as *Q* = 0.95.

**Figure 9:**
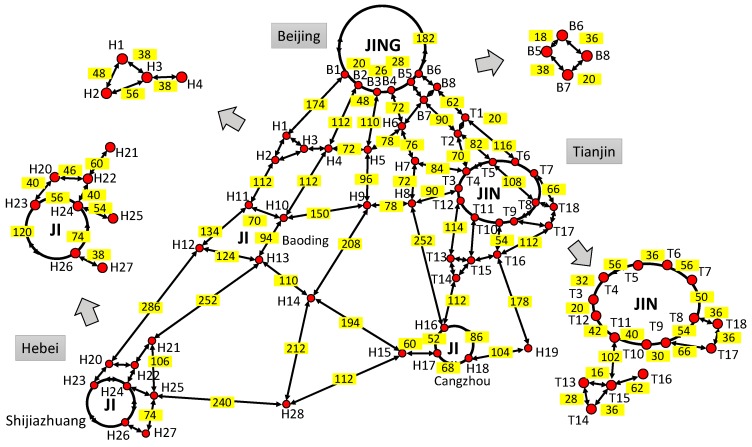
Final optimal number of sensors on each two-way segment of the numerical example.

In Figure 8b, the network nodes are the key points in the freeway network, which reflect e importation traffic flow information and will locate sensors with a high priority. As mentioned in Section 4, Case A, C, and E will be the appropriate scenarios in this network. Then, the optimal sensor location strategy of each segment will be calculated with corresponding SICF. Based on Table 1 and the models of Case A, C, and E, the optimal number of sensors located in each segment (except the network nodes) is shown in Table 3. The results show that the proposed models can deal with the SLP with various conditions in road networks and give corresponding optimal sensor layout strategies for each segment. Meanwhile, different SICFs, which reflect different spatial distribution characteristics of traffic information credibility, will lead to different sensor layout strategies. Based on Figure 8b and Table 3, the final optimal number of sensors on each two-way segment can be shown in Figure 9. In Figure 9, each solid circle means one final sensor location in network nodes which may include several monitoring sections; each number in a rectangle mean the optimal number of monitoring sections on each two-way segment.

**Figure 10:**
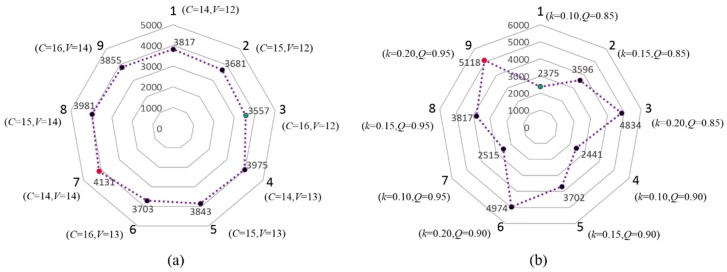
Variations of total optimal number of sensors for all segments in the freeway network with model parameters changing: (**a**) the sensitivity of parameters *C* and *V*; and (**b**) the sensitivity of parameters *k* and *Q*.

**Figure 11:**
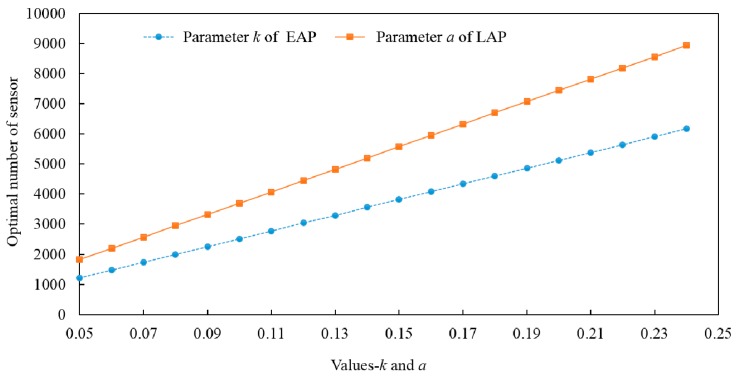
The trends of optimal number of sensors with different values of parameters *k* and *a*.

In order to analyze the sensitivity of the key model parameters, Figure 10 shows the variations of total optimal number of sensors for all segments in the freeway network with model parameters changing based on EAF models. In Figure 10a (The units of *C* and *V* are the same with Table 2), it can be drawn that parameters *C* and *V* have opposite influence patterns on the optimal number of sensors. Moreover, as the value of *C* increasing (such as points 1 → 2 → 3), the optimal number of sensors will decrease which therefore leads to low layout density of sensors. However, with the value of *V* increasing (such as points 1 → 4 → 7), a high layout density of sensor in the freeway network will be necessary. In Figure 10b, the parameters of *k* and *Q* have the same influence patterns on the optimal number or density of sensors. A bigger *Q* means more benefits from each sensor can be got, and a bigger *k* means a faster attenuation speed of traffic information credibility along the one-way road, which both mean more sensors should be deployed to obtain sufficient information and benefits. Parameters *k* and *a* reflect different spatial attenuation modes of traffic information credibility. Figure 11 shows the optimal number of sensors in the freeway network with different values of *k* and *a* based on EAF and LAF models. No matter which attenuation pattern the SICF is, as the attenuation speed of traffic information credibility increases, more sensors should be deployed to make up the lost information.

Expansibility of the models proposed in this paper is feasible for road networks. As shown in the numerical example, the maximum benefit model will be workable and effective to address the network SLP. The optimization object of the maximum benefit model is for a road segment (such as a segment between two intersections, a segment of a freeway corridor). A road network can be split into a set of segments. Given corresponding model parameters (such as *f*(*x*), *Q*, *C*, *V*, *k*, *a*, *p*_1_, *p*_2_, and *q*_1_), the optimal sensor locations of each segment in the road network can be determined. As the independence of optimal sensor locations of a one-way road, the optimal results are unrelated to other roads without consideration of the road correlation. If the optimal sensor locations of each segment are obtained in the road network, then the optimal network SLP will be determined as well. In practice, there might be correlative between two segments in a freeway corridor or a small road network. However the strength of correlation varies with time. If the strength of correlation between two segments always exceeds a reference value, just one of the two segments will be deployed by sensors; otherwise, for more common scenarios (large-scale freeway networks or urban road networks), the sensors should be deployed at their optimal locations for each segment.

## 5. Conclusions and Application Prospects

Three spatial distributions of traffic information credibility and their sensor information credibility functions proposed in this paper are helpful for understanding the data property derived from the fixed sensors, the coverage modality of traffic information along a one-way road, and the essence of the SLP. The definitions of traffic information credibility and sensor information credibility function help us consider the SLP in a novel way. Moreover, according to the theoretical formulas and numerical example for solving SLP, this paper has proven the effectiveness and availability of the maximum benefit model and its modified models. Using these models, the optimal numbers of sensors and locations with different sensor information credibility functions can be determined which are important in actual SLP projects.

The formulas for the optimal sensor locations of different cases can be using to design the sensor network structure for traffic information acquisition systems, and these formulas could also provide schemes for selecting sensor locations. Because the sensors in traffic information acquisition systems have an optimal layout mode, the structure of sensor network cannot be optional and disorganized, which illustrates that the organized structure of sensor network will be more suitable for traffic information acquisition systems. The kind of sensor networks can use fewer sensors to acquire more information and cover more areas of interest than the ones with disorganized structure.

With regard to potential application scenarios, the results of this paper could be used in the following ways: (1) for optimization of the topology structure of sensor networks for traffic information acquisition; (2) for determination of data fusion schemes with existing topology structures of sensor networks; and (3) for designing spatial characteristic-related criteria to assign the degree of contribution of a physical sensor to a point of interest, which is taken as a virtual sensor with the output determined by other physically deployed sensors. Further studies will focus on the empirical study of different spatial distributions and implement field experiments to extend the given models. Through analyzing the field or simulation data of traffic information, the spatial heterogeneity of traffic information based on the fixed sensors could be revealed. Moreover, some numerical and filed examples with more complex models and scenarios (such as urban road networks, large-scope freeway networks) would be helpful to study the SLP, which is also an important direction for future studies.

The paper is mainly concerned with the SLP of fixed sensors, which are good at whole-day, long-term traffic information acquisition. Actually, more and more non-point sensors and mobile sensors exist in modern traffic systems. These sensors are good at dynamic, flexible, real-time traffic information acquisition. Then, the non-point sensors and mobile sensors are effective supplements for traffic SLP, which, to some extent, can reduce the deployment density of fixed sensors. Studying the relationship of data from fixed sensors and non-point/mobile sensors is another significant issue.

## Figures and Tables

**Table 1:** Formulas of optimal sensor locations for different Cases and SICFs.

Cases	EAF	LAF	Two-SAF
A, C, E	*X_i_* = (*i* − 1)*d*_opt_ = (*i* − 1)*L*/(*N* − 1) *i* = 1, 2, ..., *N*.		
B, D, F	*X_i_* = *r* + (*i* − 1)*d*_opt_ = *L*/(2*n*) + (*i* − 1)*L*/*N* *=* (2*i* − 1)*L*/(2*N*), *i* = 1, 2, ..., *N*. Specifically, when *n* = 1, *X*_1_ = *L*/2.		

**Table 2:** The basic information and model parameters (Unit: *L*, km; *V*, million RMB; *C*, thousand RMB).

Index	Name	Segment	*L*	SICF	*V*	*C*	Index	Name	Segment	*L*	SICF	*V*	*C*
1	B-R6	B1 ↔ B2	8.1	SAF	18	18	46	G18	T16 ↔ H19	62.1	LAF	14	16
2	B-R6	B1 ↔ B6	73.5	SAF	18	18	47	G25	T17 ↔ T18	12.5	EAF	14	16
3	G5	B1 ↔ H1	56.9	LAF	18	18	48	G5	H1 ↔ H2	16.7	EAF	12	14
4	B-R6	B2 ↔ B3	19.5	SAF	18	18	49	G95	H1 ↔ H3	13.1	EAF	12	14
5	G5	B2 ↔ H4	36.8	LAF	18	18	50	G9511	H2 ↔ H3	20.1	EAF	12	14
6	B-R6	B3 ↔ B4	10.5	SAF	18	18	51	G5	H2 ↔ H11	35.2	EAF	12	14
7	G45	B3 ↔ H5	36.3	LAF	18	18	52	S24	H3 ↔ H4	13.7	EAF	12	14
8	B-R6	B4 ↔ B5	11.3	SAF	18	18	53	S24	H4 ↔ H5	25.1	EAF	12	14
9	G3	B4 ↔ H6	23.5	LAF	18	18	54	G4	H4 ↔ H10	48.7	EAF	12	14
10	B-R6	B5 ↔ B6	7.8	SAF	18	18	55	S24	H5 ↔ H6	26.7	EAF	12	14
11	G2	B5 ↔ B7	12.6	EAF	18	18	56	G45	H5 ↔ H9	32.8	EAF	12	14
12	S15	B6 ↔ B8	12	EAF	18	18	57	G3	H6 ↔ H7	26.3	EAF	12	14
13	G95	B7 ↔ B8	6.7	EAF	18	18	58	S3	H7 ↔ H8	25.1	EAF	12	14
14	AH3	B7 ↔ T2	29.2	LAF	18	18	59	G18	H8 ↔ H9	26.6	EAF	12	14
15	G95	B7 ↔ H6	13.7	LAF	18	18	60	S3	H8 ↔ H16	85.5	EAF	12	14
16	S15	B8 ↔ T1	24.4	LAF	18	18	61	G18	H9 ↔ H10	50.8	EAF	12	14
17	G3	T1 ↔ T2	7.3	EAF	14	16	62	G45	H9 ↔ H14	70.4	EAF	12	14
18	S30	T1 ↔ T6	39.2	EAF	14	16	63	S52	H10 ↔ H11	24.3	EAF	12	14
19	G2	T2 ↔ T4	23.8	EAF	14	16	64	G18	H10 ↔ H13	32	EAF	12	14
20	AH3	T2 ↔ T5	27.9	EAF	14	16	65	G5	H11 ↔ H12	45.8	EAF	12	14
21	G2	T3 ↔ T4	11.2	EAF	14	16	66	G107	H12 ↔ H13	42.6	EAF	12	14
22	G18	T3 ↔ T12	7.2	EAF	14	16	67	G5	H12 ↔ H20	96.5	EAF	12	14
23	G18	T3 ↔ H8	31.8	LAF	14	16	68	G107	H13 ↔ H14	37.5	EAF	12	14
24	G2501	T4 ↔ T5	19	EAF	14	16	69	G4	H13 ↔ H21	85.2	EAF	12	14
25	G3	T4 ↔ H7	29.5	LAF	14	16	70	G107	H14 ↔ H15	65.8	EAF	12	14
26	G2501	T5 ↔ T6	12.6	EAF	14	16	71	G45	H14 ↔ H28	71.9	EAF	12	14
27	AH3	T5 ↔ T8	36.7	EAF	14	16	72	G307	H15 ↔ H17	21.1	EAF	12	14
28	S30	T6 ↔ T7	19.4	EAF	14	16	73	G1811	H15 ↔ H28	82	EAF	12	14
29	S51	T7 ↔ T8	17.6	EAF	14	16	74	G3	H16 ↔ H17	17.9	EAF	12	14
30	S30	T7 ↔ T18	22.6	EAF	14	16	75	S3	H16 ↔ H18	29.8	EAF	12	14
31	S51	T8 ↔ T9	18.8	EAF	14	16	76	G1811	H17 ↔ H18	23.6	EAF	12	14
32	AH3	T8 ↔ T18	12.9	EAF	14	16	77	G1811	H18 ↔ H19	35.5	EAF	12	14
33	S50	T9 ↔ T10	10.6	EAF	14	16	78	S-Ring	H20 ↔ H22	15.8	EAF	12	14
34	S50	T9 ↔ T17	22.5	EAF	14	16	79	G5	H20 ↔ H23	14	EAF	12	14
35	S50	T10 ↔ T11	13.9	EAF	14	16	80	S9902	H21 ↔ H22	20.9	EAF	12	14
36	G18	T10 ↔ T16	18.9	EAF	14	16	81	G4	H21 ↔ H25	36.1	EAF	12	14
37	S50	T11 ↔ T12	14.8	EAF	14	16	82	S9902	H22 ↔ H24	14	EAF	12	14
38	S6	T11 ↔ T15	34.6	EAF	14	16	83	G1811	H23 ↔ H24	19.4	EAF	12	14
39	G3	T12 ↔ T13	38.6	EAF	14	16	84	G20	H23 ↔ H26	40.8	EAF	12	14
40	G104	T13 ↔ T14	9.8	EAF	14	16	85	G1811	H24 ↔ H25	18.7	EAF	12	14
41	S60	T13 ↔ T15	6	EAF	14	16	86	S9902	H24 ↔ H26	25.2	EAF	12	14
42	S6	T14 ↔ T15	12.6	EAF	14	16	87	G4	H25 ↔ H27	25.7	EAF	12	14
43	G3	T14 ↔ H16	33.1	LAF	14	16	88	G1811	H25 ↔ H28	81.4	EAF	12	14
44	S60	T15 ↔ T16	21.3	EAF	14	16	89	G20	H26 ↔ H27	13.4	EAF	12	14
45	G25	T16 ↔ T17	37.6	EAF	14	16							

**Table 3:** Optimal number of sensors in each segment.

Index	Segment	*L*	*N*_opt_^1^	Index	Segment	*L*	*N*_opt_^1^	Index	Segment	*L*	*N*_opt_^1^
1	B1 ↔ B2	8.1	10	31	T8 ↔ T9	18.8	27	61	H9 ↔ H10	50.8	75
2	B1 ↔ B6	73.5	91	32	T8 ↔ T18	12.9	18	62	H9 ↔ H14	70.4	104
3	B1 ↔ H1	56.9	87	33	T9 ↔ T10	10.6	15	63	H10 ↔ H11	24.3	35
4	B2 ↔ B3	19.5	24	34	T9 ↔ T17	22.5	33	64	H10 ↔ H13	32	47
5	B2 ↔ H4	36.8	56	35	T10 ↔ T11	13.9	20	65	H11 ↔ H12	45.8	67
6	B3 ↔ B4	10.5	13	36	T10 ↔ T16	18.9	27	66	H12 ↔ H13	42.6	62
7	B3 ↔ H5	36.3	55	37	T11 ↔ T12	14.8	21	67	H12 ↔ H20	96.5	143
8	B4 ↔ B5	11.3	14	38	T11 ↔ T15	34.6	51	68	H13 ↔ H14	37.5	55
9	B4 ↔ H6	23.5	36	39	T12 ↔ T13	38.6	57	69	H13 ↔ H21	85.2	126
10	B5 ↔ B6	7.8	9	40	T13 ↔ T14	9.8	14	70	H14 ↔ H15	65.8	97
11	B5 ↔ B7	12.6	19	41	T13 ↔ T15	6	8	71	H14 ↔ H28	71.9	106
12	B6 ↔ B8	12	18	42	T14 ↔ T15	12.6	18	72	H15 ↔ H17	21.1	30
13	B7 ↔ B8	6.7	10	43	T14 ↔ H16	33.1	47	73	H15 ↔ H28	82	121
14	B7 ↔ T2	29.2	45	44	T15 ↔ T16	21.3	31	74	H16 ↔ H17	17.9	26
15	B7 ↔ H6	13.7	21	45	T16 ↔ T17	37.6	56	75	H16 ↔ H18	29.8	43
16	B8 ↔ T1	24.4	37	46	T16 ↔ H19	62.1	89	76	H17 ↔ H18	23.6	34
17	T1 ↔ T2	7.3	10	47	T17 ↔ T18	12.5	18	77	H18 ↔ H19	35.5	52
18	T1 ↔ T6	39.2	58	48	H1 ↔ H2	16.7	24	78	H20 ↔ H22	15.8	23
19	T2 ↔ T4	23.8	35	49	H1 ↔ H3	13.1	19	79	H20 ↔ H23	14	20
20	T2 ↔ T5	27.9	41	50	H2 ↔ H3	20.1	29	80	H21 ↔ H22	20.9	30
21	T3 ↔ T4	11.2	16	51	H2 ↔ H11	35.2	51	81	H21 ↔ H25	36.1	53
22	T3 ↔ T12	7.2	10	52	H3 ↔ H4	13.7	19	82	H22 ↔ H24	14	20
23	T3 ↔ H8	31.8	45	53	H4 ↔ H5	25.1	36	83	H23 ↔ H24	19.4	28
24	T4 ↔ T5	19	28	54	H4 ↔ H10	48.7	71	84	H23 ↔ H26	40.8	60
25	T4 ↔ H7	29.5	42	55	H5 ↔ H6	26.7	39	85	H24 ↔ H25	18.7	27
26	T5 ↔ T6	12.6	18	56	H5 ↔ H9	32.8	48	86	H24 ↔ H26	25.2	37
27	T5 ↔ T8	36.7	54	57	H6 ↔ H7	26.3	38	87	H25 ↔ H27	25.7	37
28	T6 ↔ T7	19.4	28	58	H7 ↔ H8	25.1	36	88	H25 ↔ H28	81.4	120
29	T7 ↔ T8	17.6	25	59	H8 ↔ H9	26.6	39	89	H26 ↔ H27	13.4	19
30	T7 ↔ T18	22.6	33	60	H8 ↔ H16	85.5	126				

^1^ The number is just the optimal number of sensors for each one-way segment; for a two-way segment, the number will be doubled, which is shown in Figure 9.
